# Oral Hygiene Practices among Adults with Intellectual Disabilities—A Pilot Study

**DOI:** 10.3390/dj10080155

**Published:** 2022-08-19

**Authors:** Muhammad Afif Hakimin Abdullah, Ishak Nurul Sa’idah, Joe Knights, Sachinjeet Kaur Sodhi Dhaliwal, Long Chiau Ming, Jagjit Singh Dhaliwal

**Affiliations:** 1 PAPRSB Institute of Health Sciences, Universiti Brunei Darussalam, Gadong BE1410, Brunei; 2 Special Care Dentistry Unit, Raja Isteri Pengiran Anak Saleha Hospital, Bandar Seri Begawan BA1712, Brunei

**Keywords:** oral hygiene, oral health, dental care for disabled, intellectual disability, Brunei

## Abstract

It is known that the oral health status of people with intellectual disabilities (IDs) is likely to be compromised as compared with the general population. Until recently, the trend of oral hygiene among the adult population with intellectual disabilities in Brunei Darussalam had yet to be studied. Thus, the aim of this study was to determine the oral hygiene practices, oral health knowledge and dental appointment patterns among the adult population with IDs in Brunei Darussalam. A cross-sectional study was conducted at different service providers in Brunei Darussalam for adults with IDs. An easy-to-read questionnaire was distributed to the participants. The responses of the questionnaire for IDs were analyzed. A total of 34 participants were recruited. It was found that all participants were practicing their daily oral hygiene routine. In terms of dental visits, more than half visit the dentist at least once a year. The majority of the participants agreed that visiting the dentist every 6 months was essential. This study showed that adults with IDs in Brunei Darussalam were aware of the importance of oral health and were maintaining their daily oral hygiene, although not with the ideal method. It is important for clinicians to not only educate the individuals with IDs but also their carer(s) when it comes to oral health knowledge, as carers play a key role in the oral health of the people under their care.

## 1. Introduction

According to the American Association on Intellectual and Developmental Disabilities, intellectual disabilities (IDs) are defined as a considerable impairment in an individual’s cognitive ability as well as in their ability to do their everyday routine and interact with people [1]. The impairment may become noticeable during their developmental phase [2]. ID has become one of the most common disabilities among children, and as a result of increased life expectancy it is predicted that the number of adults with IDs will continue to grow [3]. An individual may have a type of ID by itself or it may also come together with congenital malformation, neurological features and/or differences in developmental behavior [4].

It was concluded in a meta-analysis study involving 52 countries that the prevalence of ID is 10.37 per 1000 population; where more males were affected among adults as well as in children [5]. A similar trend can also be observed for individuals with disabilities in Brunei Darussalam [6]. In 2016, it was reported that there were approximately 9282 individuals with disabilities in Brunei Darussalam. However, it was not specified how many of them had IDs [7]. One of the most common conditions with ID is Down syndrome (DS), where the individuals possess an extra copy of chromosome 21 resulting in a trisomy [8]. Individuals with DS are typically characterized by intellectual disabilities, physical abnormalities and are often linked with various medical complications, such as congenital heart disease and Alzheimer’s disease [9]. They may also be identified through physical features such as a protruding tongue due to macroglossia, single palmar crease, up-slanting palpebral fissure, flat nasal bridge, low muscle tone and small chin [10].

The oral health of an individual with IDs is an important aspect of their overall condition as it can affect their daily life and general health [11,12,13]. The oral health status of people with intellectual disabilities (IDs) is likely to be poorer compared to the general population. It was deduced that people with IDs tend to have more missing teeth, untreated caries as well as more dental extractions compared to restorations [14,15,16]. It has been concluded from various studies that individuals with IDs have either poorer oral hygiene compared to the general population or no difference at all [17]. However, in a study done among 33 individuals with DS about 42.4% and 21.2% were reported to have very good oral hygiene and good oral hygiene, respectively; while the remaining percentage was reported to have poor or very poor oral hygiene [18].

Meanwhile, autism spectrum disorder (ASD), similar to IDs, is a developmental disorder that is often characterized by an unchanging order of behavior and interests, as well as trouble with interaction and socializing with other people [19,20]. About 20–50% of individuals with ASD are often linked with an intellectual disability [21]. Although ASD itself may not have a direct link with any types of oral diseases, it may come about as a consequence of impaired behavior, hyposensitivity to pain, certain dietary or eating patterns as well as negligence toward oral health [22,23]. Similar to DS, individuals with ASD are also linked with a higher prevalence of periodontal diseases [24,25].

In reference [26], the trend of oral hygiene practice among adults with IDs is yet to be studied and it is important to keep watch on this as oral health conditions are positively linked with the degree of disability and age in people with IDs [14,15,27]. The present study aimed to determine the oral hygiene practices and oral health knowledge among the adult population with IDs in Brunei Darussalam. The pattern of dental appointments of this group of population was also studied to understand their visit adherence.

## 2. Methods

### 2.1. Study Design, Population and Sample

This research was a cross-sectional study, where information regarding oral hygiene practices, oral health knowledge and dental visiting trend among adults with IDs was obtained with the use of questionnaires. Participants were selected from four service providers for the adult population with IDs in Brunei Darussalam.

### 2.2. Eligibility Criteria

The inclusion criteria of this study were adults with IDs who were 18 years old and above and had been clinically diagnosed by a trained pediatrician related to the status of IDs. Individuals who were from a service provider located outside of Brunei-Muara district were excluded from this study.

### 2.3. Questionnaire

A systematic literature review was carried out to search for relevant literature and a single questionnaire that may answer the aims of this research. Following the review, there was no single questionnaire that met the requirement. Therefore, the questionnaire that was used in this research project was adapted from a similar unpublished report on children with disabilities in Brunei Darussalam [26]. The questionnaire was first reviewed by three senior dentistry academicians and three senior carers of people with IDs. These panelists reviewed the questionnaire based on content simplicity, relevance and ambiguity. Further modification of the questionnaire draft, based on the collected comments, was made.

People with IDs have different communication, reading and decision-making abilities. Hence, an easy-to-read format was developed for the participants’ information sheet (PIS), consent forms and questionnaires. This included the use of photos to maximize participants’ ability to understand the study, support their decision-making capacity and understand and answer the questions themselves.

### 2.4. Data Collection

The researcher (via the assistant registrar of the institute) mailed letters seeking access to the managing directors of the selected service providers to obtain permission to carry out the research at their respective centers on selected dates. An information pack that contained the participant information sheet, consent form and poster regarding the details of the study was distributed by the gatekeeper from each center to participants who fulfilled the inclusion criteria. Any interested adults with IDs and their caregivers informed the gatekeeper of whether they were keen on participating. Only those who had given informed consent and signed the consent form were included in this study. The information pack was given two weeks prior to the selected day of data collection to give time for eligible participants to make their decision.

On the day of data collection, the questionnaire was given to recruited participants after recording their consent. The principal researcher and one of the supervisors were present during the data collection to answer any queries. The participants were given at least 15 min to answer the questionnaire. No time limit was given to allow the adults with IDs to answer the questionnaire at their own pace. Caregivers or teachers were present to support participants in reading, understanding and answering the questionnaire. If they were not able to understand the questions at all, the participant’s caregiver could answer the questionnaire for the participant. Completed questionnaires were collected by the researchers.

Ethics approval was granted by the joint Ethics Committee of the PAPRSB Institute of Health Sciences (IHSREC) and the Medical and Health Research Ethics Committee (MHREC), Ministry of Health, Brunei Darussalam.

### 2.5. Data Analysis

The data acquired from the questionnaire were inserted and arranged in MS Excel for analysis with the use of RStudio software. Descriptive statistical analysis was applied to the collected data.

## 3. Results

### 3.1. Sociodemographic

A total of 34 participants were obtained throughout the data collection period of this study. The mean (SD) age of participants in this study was 24.3 (5.4) years. Among the 34 participants, 22 (64.7%) were males and the remaining 12 (35.3%) were females. With regard to the different types of intellectual disabilities, 4 (11.8%) were participants with Down syndrome, 11 (32.3%) were participants with autism and 19 (55.9%) were “Others”, which mainly comprised delayed speech development (Table 1).

### 3.2. Oral Hygiene Practices

All the participants reported that they cleaned their teeth using toothbrushes and toothpaste. In terms of brushing frequency, it was found that the majority (52.6%) of participants under the “Others” type of intellectual disabilities brushed more than twice in a day. It was observed that the combined tooth-brushing method (both horizontal and vertical method combined) is preferred by participants of the three different types of IDs and it can be seen that all (100%) DS participants, the majority of ASD participants (63.6%) and “Others” type of IDs (52.6%) were using this method in their daily oral hygiene practice (Table 2).

Furthermore, it was found that 24 (70.6%) of the 34 participants practiced tongue brushing in their daily oral hygiene. In addition, the participants were also asked whether they had noticed any smell from their mouths. It was seen that among the 11 participants who noticed four (36.4%) and seven (63.6%) were participants with ASD and under the “Others” type of IDs, respectively (Table 2).

### 3.3. Dental Visits Trend

In terms of dental visits, it was observed that 14 (41.2%) of the participants reported that they go to the dentist only when they are experiencing a problem. As for participants with DS, three (75%) reported that they visit the dentist once in six months and the remainder visits when experiencing a problem. Among participants with ASD, four (36.3%) reported they visit once in three months, three (27.3%) visit once between one and two years, two (12.2%) visit once in six months and two (12.2%) visit only when they have a problem. Upon analysis of the dental visiting trend between genders, it was found that nine (40.9%) male participants and five (41.7%) female participants only visit when experiencing problems (Table 2).

### 3.4. Oral Health Knowledge

Regarding oral health knowledge, 27 (79.4%) of the participants agreed that it is important to meet a dentist every six months. Ironically, although the “Others” category had the highest percentage in agreement on this statement, eleven (57.9%) reported they only visit the dentist when there is a problem. Other than that, 22 (64.7%) of all participants reported that they are aware that oral health is linked to systemic health (Table 2).

### 3.5. Method of Answering Questionnaire

Interestingly, it was found that more than half (52.9%) of the questionnaires were answered by the participants themselves and fewer than half (35.3%) of the questionnaires were answered by caregivers. While the remaining (11.8%) questionnaires were answered by participants with assistance from caregivers (Table 2).

## 4. Discussion

### 4.1. Statement of Principal Findings

This study aimed to obtain insights into the current trend of oral hygiene practices, dental visit trends as well as oral health knowledge of adults with IDs.

The findings showed that all participants reported maintaining their daily oral hygiene, although not all were practicing it in an ideal way. It is recommended by experts to brush twice a day for 2–3 min using the modified bass technique [28,29,30]. It may be due to initiatives made by the center itself or the government to emphasize the importance of oral health. In conjunction with World Oral Health Day, the Ministry of Health Brunei dental services had put in efforts to increase awareness of oral hygiene practices and oral health to the general public [31]. For example, in the oral health promotion booklet 2014 there is information about the importance and maintenance of oral hygiene, tooth-brushing methods as well as applications of fluoride varnish [32].

Among the three categories of IDs, it was seen that participants with ASD have the highest proportion of not brushing their tongue. This might be due to their clinical nature of being hypersensitive to touch [33]. It is thought that the primary source of halitosis is from the back of the tongue [34], which is why it is recommended to brush twice daily along with tongue cleaning to manage halitosis [35]. However, in this study, more than half of participants with ASD reported that they had never noticed any bad odor from their oral cavity even though more than half of the participants reported not brushing their tongue.

Like any other specialist departments in Brunei Darussalam, visits to the dentist are appointment-based, where the population with the greatest disease burden would have their consecutive appointments closer to each other. However, a little less than half reported visiting the dentist only when experiencing a problem. A barrier that may hinder these participants from getting regular dental check-ups may be odontophobia [36]. Women are commonly reported to be more dentally anxious compared to men [37]. However, in this study, the proportion of males and females that visited the dentist when experiencing dental problems was more or less the same. Other than odontophobia, it is probable that there are other factors that had led to this finding, for example, oral health awareness of their caregivers and socio-economic factors. Based on a study done by Shah et al., 55% of special needs caregivers reported that they would only visit the dentist in case of a problem [38].

In terms of a DS individual’s oral health, it was reported that they have a higher rate of periodontal disease compared to individuals without DS [39]. This is thought to be due to one of the complications of having DS, which is a compromised immune system. This may increase the susceptibility to infections [40]. In addition, DS individuals often have delayed dental eruption, a non-existing frontal or maxillary sinus, a thick hard palate on each side that may affect mastication and speech and a hypoplastic mid-facial region that affects the maxilla, bridge of the nose and bones in the region [10].

### 4.2. Findings Compared with Previous Studies

Firstly, in terms of oral hygiene practices, a similar trend was observed when comparing the results with the results of another study in Brunei, whereby the majority of the participants were practicing their daily oral hygiene [26]. In a study published by Porovic et al., it was seen that more than half of the participants with DS possessed good oral hygiene [18]. This further corroborates the findings of this study concerning oral hygiene practices. Chlorhexidine has been very helpful in patients with compromised dental status [41].

In a study reported by Stein et al., 57% of participants with ASD did not like the feeling of having a toothbrush in their mouth [33]; these findings are concurrent with the findings of this study, as participants with ASD in this study had the highest percentage of not brushing their tongue.

In terms of the dental visiting trend, fewer than half of the participants in this study reported that they visit the dentist at least twice a year. Similarly, this trend can also be seen in the study on the younger population with IDs in Brunei [26]. However, another study reported that only a small percentage of young adults with IDs visit the dentist at least twice a year [42].

In terms of oral health knowledge, the majority of the participants in this study agreed that visiting the dentist every six months is important. However, in a similar study on the younger population, it was found that only less than half agreed to the same statement. This may be due to the difference in age as well as different proportions of conditions of IDs, hence different perceptions. As individuals with IDs are generally considered a vulnerable population, there have not been many studies on this population due to ethical reasons.

### 4.3. Strengths and Limitations

One of the strengths of this study is the development of easy-to-read text in all documents to maximize the participants’ capacity for understanding the text. Sufficient time was also provided to the participants to make decisions and during data collection. Oral health research data comparisons can be carried out as the questionnaire used was the same as a questionnaire from a similar study on the younger population with IDs in Brunei Darussalam.

In terms of limitations, only a small sample size was obtained due to time limitations and the sensitivity of the topic. In addition to the time limitation, the COVID-19 outbreak in the country had also led to the decision to stop the data collection period earlier than planned. Furthermore, the severity of each participants’ ID was not assessed, which could be a potential factor for negligence in maintaining good oral health.

Another limitation is in the participants’ bias, which may be related to the nature of self-reporting. Hence, this may affect the results of the findings and the accuracy of the trends. The questionnaire used was adapted from an existing questionnaire due to time limitations.

## 5. Conclusions

Valuable data were obtained, as research on this topic had never before been carried out on this population in Brunei Darussalam. More research on this topic may enable clinicians and policymakers to gaining further insights into the current trends to facilitate improved initiatives and interventions. These can then improve the oral hygiene practices, oral health knowledge and awareness of this population. It is important for clinicians to not only educate the individuals with IDs but also their carer(s) when it comes to oral health knowledge, as carers play such a key role in the oral health of the people under their care.

This study showed that adults with IDs in Brunei Darussalam were aware of the importance of oral health and were maintaining their daily oral hygiene, although not with the ideal method. However, the trend of dental visits was still lacking, which may be affected by internal and external factors.

## Figures and Tables

**Table 1:** Sociodemographic of participants (*n* = 34).

Sociodemographic		Total (*n* = 34)		Mean	(SD)
		*n*	%		
Age				24.3	(5.4)
Gender					
	Male	22	(64.7)		
	Female	12	(35.3)		
Type of Intellectual Disabilities					
	Down Syndrome	4	(11.8)		
	Autism	11	(32.3)		
	Others	19	(55.9)		

*n* = Frequency; % = Frequency Percentage; SD = Standard Deviation.

**Table 2:** Questions and Responses of Participants According to Their Medical Condition.

Questionnaire	Type of Intellectual Disability			%
	Down Syndrome *n* (%)	Autism Spectrum Disorder *n* (%)	Others *n* (%)	
1. Do you clean your teeth?				
Yes	4 (11.8)	11 (32.3)	19 (55.9)	100
No	0 (0.0)	0 (0.0)	0 (0.0)	0
2. How do you clean your teeth?				
Toothbrush and toothpaste	4 (11.8)	11 (32.3)	19 (55.9)	100
Toothbrush and powder	0 (0.0)	0 (0.0)	0 (0.0)	0.0
Others (Datun, Finger, Charcoal powder)	0 (0.0)	0 (0.0)	0 (0.0)	0.0
3. How often do you brush your teeth each day?				
Once	0	2 (40.0)	3 (60.0)	14.7
Twice	3 (20.0)	6 (40.0)	6 (40.0)	44.1
More than twice	1 (7.2)	3 (21.4)	10 (71.4)	41.2
Sometimes	0 (0.0)	0 (0.0)	0 (0.0)	0.0
4. What type of tooth-brushing methods do you use?				
Vertically	0 (0.0)	1 (14.3)	6 (85.7)	20.6
Horizontally	0 (0.0)	3 (30.0)	3 (30.0)	17.6
Combined	4 (0.0)	7 (41.2)	10 (58.8)	61.8
5. Which other methods do you use for cleaning your teeth (plaque control)? (Can tick more than 1 box) *n*^a^ = 36				
Dental floss	0 (0.0)	3 (75.0)	1 (25.0)	11.1
Interdental brushes	0 (0.0)	2 (33.3)	4 (66.7)	16.7
Toothpicks	0 (0.0)	3 (42.9)	4 (57.1)	19.4
None	4 (21.1)	5 (26.3)	10 (52.6)	52.8
6. Do you clean your tongue?				
Yes	3 (12.5)	6 (25)	15 (62.5)	70.6
No	1 (10.0)	5 (50.0)	4 (40.0)	29.4
7. Have you ever noticed smell from your mouth?				
Yes	0 (0.0)	4 (36.4)	7 (63.6)	32.4
No	4 (17.4)	7 (30.4)	12 (52.2)	67.6
8. Do you know that oral health is related to systemic health?				
Yes	3 (13.6)	7 (31.8)	12 (54.6)	64.7
No	1 (8.3)	4 (33.3)	7 (58.3)	35.3
9. How often do you visit a dentist?				
Only when problem	1 (7.1)	2 (14.3)	11 (78.6)	41.2
Once in 3 months	0 (0.0)	4 (44.4)	5 (55.6)	26.5
Once in 6 months	3 (37.5)	2 (25)	3 (37.5)	23.5
Between 1 and 2 years	0 (0.0)	3 (100)	0 (0.0)	8.8
10. Do you think it is essential to meet a dentist every 6 months?				
Yes	3 (11.1)	10 (37.0)	14 (51.9)	79.4
No	1 (14.3)	1 (13.3)	5 (71.4)	20.6
11. How did you answer this questionnaire?				
I answered this myself	1 (5.5)	5 (27.8)	12 (66.7)	52.9
Assisted by caregiver	1 (25.0)	2 (50.0)	1 (25.0)	11.8
Answered by caregiver	2 (16.7)	4 (33.3)	6 (50.0)	35.3

*n* = Frequency of Cases; % = Frequency Percentage; a = Frequency of Responses.

## Data Availability

Data will be provided on request.
